# Frugivory in Canopy Plants in a Western Amazonian Forest: Dispersal Systems, Phylogenetic Ensembles and Keystone Plants

**DOI:** 10.1371/journal.pone.0140751

**Published:** 2015-10-22

**Authors:** Pablo R. Stevenson, Andrés Link, Sebastian González-Caro, María Fernanda Torres-Jiménez

**Affiliations:** 1 Departamento de Ciencias Biológicas, Universidad de Los Andes, Bogotá, Colombia; 2 Departamento de Ciencias Biológicas & Facultad de Administración, Universidad de Los Andes, Bogotá, Colombia; 3 Ecosystem Services and Climate Change Lab, Medellin Botanical Garden, Medellín, Colombia; 4 Institute of Evolutionary Biology, The University of Edinburgh, Edinburgh, United Kingdom; University of Tartu, ESTONIA

## Abstract

Frugivory is a widespread mutualistic interaction in which frugivores obtain nutritional resources while favoring plant recruitment through their seed dispersal services. Nonetheless, how these complex interactions are organized in diverse communities, such as tropical forests, is not fully understood. In this study we evaluated the existence of plant-frugivore sub-assemblages and their phylogenetic organization in an undisturbed western Amazonian forest in Colombia. We also explored for potential keystone plants, based on network analyses and an estimate of the amount of fruit going from plants to frugivores. We carried out diurnal observations on 73 canopy plant species during a period of two years. During focal tree sampling, we recorded frugivore identity, the duration of each individual visit, and feeding rates. We did not find support for the existence of sub assemblages, such as specialized vs. generalized dispersal systems. Visitation rates on the vast majority of canopy species were associated with the relative abundance of frugivores, in which ateline monkeys (i.e. *Lagothrix* and *Ateles*) played the most important roles. All fruiting plants were visited by a variety of frugivores and the phylogenetic assemblage was random in more than 67% of the cases. In cases of aggregation, the plant species were consumed by only primates or only birds, and filters were associated with fruit protection and likely chemical content. Plants suggested as keystone species based on the amount of pulp going from plants to frugivores differ from those suggested based on network approaches. Our results suggest that in tropical forests most tree-frugivore interactions are generalized, and abundance should be taken into account when assessing the most important plants for frugivores.

## Introduction

Interactions leading to the escape of seeds from parental plants and the colonization of adequate sites for recruitment play a relevant role in dynamics of many ecosystems [1, 2, 3]. Evidence supports that biotic interactions such as negative density dependence mediated by predators and pathogens play a major role determining the chances of plant establishment [4, 5]. For instance, seed dispersal usually increases plant fitness by avoiding enemies or from the benefits of colonizing adequate recruitment sites [1]. In addition, light is a limiting factor for seedling recruitment in closed canopy forests, favoring the evolution of large seeds [6], whose larger reserves make them more resistant to herbivore attacks [7–9]. Furthermore, the frequency of large seeded plants is higher in infertile than in fertile soils [10] and wet tropical forests tend to have leached soils. In spite of the advantage of large-seeded species in tropical forests [11] there seems to be a trade-off between seed size and the availability of potential dispersal agents [12] since seed dispersal for large seeded plants is often restricted to scatter-hoarder rodents and to large frugivores [13–16]. Not surprisingly, plants in natural tropical forests are predominantly dispersed by animal vectors [17–18]. In spite of this pattern, there is great variation in the frequency and relative importance of different dispersal agents within [19–20] and across environments [17–18] and we currently do not fully understand how these assemblages originated, how they operate, and the relative importance of their components.

The first hypothesis on the evolution of plant-frugivore interactions envisioned a strong role of selective forces imposed by animal agents, in such a way that many fruit traits (i.e. color, accessibility, size, defenses and nutritional contents) evolved to attract particular dispersal agents [21]. These ideas were the basis for the formulation of the evolution of specialized vs. generalized dispersal systems [22–24]. Thus, according to this hypothesis, plant-frugivore interactions should be discriminated in two groups, specialized and generalized. Specialized plants should show larger seeds, lower fecundity, extended crops, higher lipid (or protein) contents, and higher removal and dispersal rates than generalized plants [25]. On the other hand, specialized frugivores (i.e. birds) should be larger, highly dependent on fruits, more efficient dispersers, and should use less plant species than generalized frugivores [25]. Some studies that have explicitly tested the existence of these dispersal systems have provided little support for this hypothesis [26–27]. However, some components of the theory (e.g. the differential treatment by frugivores in some circumstances) have been supported [28–29]. No study in tropical forests has examined yet the existence of these specialized and generalized dispersal systems at the community level; however, the degree of specialization has been calculated in few studies. For instance, in a tropical forest in Kenya, it was found that plant-frugivore interactions in the canopy were less specialized than in lower forest strata [30].

Some morphological fruit traits have evolved in association, as a consequence of being dispersed by a particular set of frugivores (i.e. seed dispersal syndromes *sensu* [21]). For instance, relatively small, unprotected and brightly colored fruits have been classified in a dispersal syndrome related to avian frugivory; while large, protected and less colorful fruits are mainly dispersed by mammals, such as primates [21]. Although these associations have been found in other tropical forests [31], their occurrence might not be general to all of them [12, 32–33]. However, other theories suggest that the evolution of fruit traits may not represent adaptation to current seed dispersal agents, but may result as ecological anachronisms of conserved fruit traits [34] or developmental consequences of the evolution of other plant traits [35].

If dispersal agents differ in their efficiency as seed dispersers [36], plants might evolve morphological or chemical traits to generate dispersal filters. However, most fruit-animal interactions tend to show little specificity and each plant species tends to interact with a large number of frugivores [37]. In addition, most plant-animal mutualisms tend to be organized in a nested fashion [38], where specialists interact with subsets of the species with which generalists interact. According to these studies, the degree of specialization in mutualistic networks should behave as a continuum and not in a bimodal way, as suggested by generalized vs. specialized dispersal systems.

One approach that has not been used to examine the relative success of plants in generating filters is the phylogenetic assemblage approach [39], which quantifies the degree of phylogenetic aggregation or dispersion in particular communities. Thus, if plants have succeeded at generating filters, and we assume that filters may affect closely related species in a similar way, then we expect to find frugivore communities with phylogenetic aggregation (i.e. only some groups of related species should feed on one particular plant). However, if plant-frugivore mutualisms tend to be generalist, one should expect that the abundance of frugivores should explain visitation patterns, as well as random patterns in the phylogenetic assemblage of frugivores. Finally, in scenarios of high competition for fruits, overdispersed patterns should be expected [39], under the assumption that related species share more resources than unrelated ones.

In terms of species conservation and ecosystem functioning, it is relevant to know which plants are more important for frugivore communities and assess their potential role as keystone species. Traditionally, keystone plants in tropical forests have been considered those producing fruits in periods of resource scarcity [40]. A more general definition considers species to be keystone when its impact on a community is large and disproportionally large compared to its abundance [41]. Some studies searching for keystone plants have adhered to this idea [42], but not others [43]. In addition, it has been suggested that the scarcity period may be less problematic for large frugivores that are able to accumulate fat reserves in their bodies than for small frugivores [43]. According to this idea, plants producing in periods of fruit abundance should also be considered as keystone plants, with the potential to severely affect frugivore populations. Recently, network approaches have considered a variety of mechanisms to quantify keystone species based on the topology of food webs [44]. These approaches usually consider a species to be keystone if it is highly connected node or a hub node connecting two or more compartments inside the network.

In order to understand the potential pressures generated by dispersal agents on fruit traits, the first step is to have some understanding of the behavior of frugivores and knowledge about which animal species use particular plants [21]. Amazingly, there are few quantitative descriptions on which frugivores visit and disperse seeds of a large array of tree species within a single forest community [45–46]. This task seems challenging because there is great variation in the role of frugivores at temporal and spatial scales [47], even in less complex environments [48]. The main aims of this study were to 1) test the existence of generalized vs. specialized dispersal systems and to determine the overall degree of specialization in the network, 2) test the occurrence of phylogenetic aggregation in frugivore assembles and 3) examine the performance of different approaches to identify keystone plants, including unweighted and weighted network indices. While unweighted indices provide information about how well a node is topologically connected, weighted indices provide information about the strength of interactions. Finally, we use an estimate of the pulp biomass actually going to the level of primary consumers (as a proxy of energy use [49]), including information of the strength of the interaction and the abundance of the plant population.

## Materials and Methods

### Study area

The study area is a tropical lowland forest on the eastern border of Tinigua National Park (201,875 ha), in the Department of Meta, Colombia (2° 40' N and 74° 10' W, 350–400 m above sea level). The main field site of the Center of Ecological Investigations La Macarena (CIEM), where this study took place, is on the west margin of the Duda River. Rainfall is markedly seasonal in the region, with a 2–3 month dry period (December to March), and average annual precipitation for the study years was 2782 mm [50]. Plant diversity is relatively high, reaching more than 100 species per hectare in *terra firme* forests [51].

### Field protocol

We carried out a total of 3343 h of diurnal observations on 73 plant species during a period of two years (1999–2001). We selected zoochorous ripe fruiting plants that had good crown visibility. Ground observation did not seem to be biased towards large frugivores; in fact, 69% of the bird species observed were small bodied (weighting less than 100 g). We limited our observations to periods of peak frugivore activity (usually between 6:00 and 10:00 h), and the total sampling time was variable among species (average = 46, range = 22–115 h). We avoided sampling during rain and we did not use blinds or camouflage, which did not seem to affect the behavior of canopy frugivores that had been subject to observation for more than one decade. However, our presence during observations probably influenced the approach and feeding by terrestrial mammals and birds such as coatis, tayras, tapirs, and trumpeters.

During fruiting tree observations we recorded the duration of each visit by every frugivore (in minutes), its identity (species), and feeding rates. Feeding rates for primates were recorded as the total number of fruits manipulated during periods of 30 s, when a focal animal was clearly visible. Focal sampling for bird species was set to periods of 10 s. We multiplied total feeding time by average feeding rates to estimate the number of fruits handled by each frugivore species. For all plant species consumed by primates, we maintained total primate-feeding time from our observations, but modified the proportion assigned to each monkey by using the relative consumption found in intensive studies of primate behavior [52], because they were based on larger datasets.

### Ethics Statement

According to the bylaws of the institutional ethics committee of the Universidad de Los Andes, we did not need any formal authorization to conduct this study, as our project did not involve animal capture, nor experimentation. We did not include known endangered plants in the study. We obtained permits (#459–96 and PIDB DTAO 007–12) from UPNNC (*Unidad de Parques Nacionales Naturales de Colombia*).

### Statistical Analyses

We constructed a community matrix where each cell X_*ij*_ represents the number of fruits of plant *j* manipulated by the frugivore *i*. We used non-metric multidimensional scaling (NMDS) to examine subsets of plant species consumed by frugivores, and a canonical analysis to explore the fruit traits associated with the ordination. In order to test some predictions regarding the hypothesis on the existence of generalized vs. specialized dispersal systems, we used a nonparametric ordination of plants, mainly based on plant traits such as seed width, % of lipids in the pulp, crop size, and crop duration [53], using the *vegan* package in R. In addition, we used simple regression models to test seven predictions from this theory: pulp quality (i.e. lipid content) should be positively associated with 1) crop duration, 2) seed size, 3) frugivore size, and negatively associated with 4) crop size, 5) visitation rate, 6) the number of visiting species, and 7) the probability to recruit below parents [25]. In addition, a non-parametric ordination based on the percentage of fruits manipulated by each frugivore species was generated to examine potential grouping of plants based on frugivore types.

To assess pulp quality per fruit, we measured the proportion of lipids. Seed width was considered as a proxy of seed size [15]. Information on crop size and duration was extracted from phenological studies including four years of phenological sampling [50]. The degrees of freedom for these analyses varied, depending mainly on the number of species with nutritional information (N = 15–39). We also used regression models to explain feeding times from the abundance of each frugivore. Primate densities were estimated using line transect methods [54]. Avian abundance was extrapolated from the relationship between the density estimates on line transects (measured for *Pipile pipile*, *Penelope jaquacu*, and *Psophia crepitans*) and the frequency of observations of bird species in the study site (n = 3075) [55].

Potential plant filters to exclude frugivores were analyzed using the phylogenetic community indices: net relatedness index and nearest taxon index (NRI and NTI, respectively). These indices measure the evolutionary distance of co-occurring species in terms of the length of connecting branches in phylogenetic trees, comparing the mean distance among all species in the assemble (NRI) or the distance among the near taxon (NTI) with a null random distribution [39]. We used a swap model to avoid the influence of species richness and frequency in our estimates. In both indices, negative values represent phylogenetic overdispersion and positive values represent phylogenetic aggregation.

The phylogeny of frugivore assemblages was reconstructed using mitochondrial DNA sequences published in GenBank. We aligned these sequences using the *Muscle* algorithm with default parameters. Then we reconstructed a phylogenetic tree using *BEAST* software. Also, we used node age estimates to calibrate our tree from previous phylogenetic studies.

On the other hand, we obtained a phylogenetic tree to plant species in our dataset from *Phylomatic* [56], using the Angiosperm Phylogeny Group’s consensus tree (R2010829). We calibrated this phylogeny based on Wilkstrom et al. [57] node dates applying *bladj* algorithm in *Phylocom* [58]. NRI and NTI were calculated in *Picante* package in R.

To explore potential keystone plant species in terms of well connected nodes or hub nodes important for network’s stability, we used the community consumption matrix (S1 Dataset) as input to compute weighted and un-weighted network indices of the bipartite network with the *Bipartite* 2.05 package from R [59]. Computed indices are based on connectivity and centrality of a node in relation to the remaining nodes in the network [60–62] and in addition ecological information was used to identify keystone species. Selected un-weighted indices consider merely structural and connective features for each species, while weighted indices consider interaction strength as the number of manipulated fruits (Table 1). For instance, closeness indices identify the shortest paths connecting one node to the rest of the nodes in the network [60]. Likewise, betweenness quantifies how many times a node is on the shortest path between all other node pairs in the network [63]. Nodes densely connected that are often intermediate between two other nodes in the network are thought to modulate and propagate stability and extinction processes within a community by being the central connection between two or more sections [64–65]. Nestedness of the binary matrix was calculated using the NODF metric (Nestedness metric based on Overlap and Decreasing Fill, [66]) and compared with the CE (row-column probability) and the ER (absolute random) models with 1000 replicates implemented in *Aninhado* 3.0 [67], and the species’ nestedness rank calculated using *Bipartite* 2.05 [59]. To simplify value interpretation, values of *d’*_*i*_, *d*, nestedness rank (NR) and species specificity index (SSP) were converted as 1-i. The specialization index for the matrix (H2’) was calculated using the *Bipartite* 2.05 package from R [59]. The resulting index was compared with the distribution of the H2’ values obtained from a set of 500 null matrices generated using the function null model under the vasnull method implemented in *Bipartite*. Using the *Mass* package for R [68], NMDS and correlation tests were performed to search for redundancy among all indices excepting NODF and H2’. Additionally, species were classified as peripheral or core species (lower and higher number of interactions compared with other nodes in the same trophic level, respectively) following Dáttilo and coworkers [69].

**Table 1:** Network centrality indices used to identify possible keystone plant species and average values and variance of each index.

Index	Information type	Mean value	Variance	Description	References
Species Degree (D)	Un-weighted	6.644	18.816	Sum of interactions per species.	[65]
Normalized Degree (ND)	Un-weighted	0.081	0.003	Scaled by the number of possible partners.	
Generality (G)	Un-weighted	0.810	1.000	Mean number of manipulating animals per plant.	[69]
Nested rank (NR)	Weighted	0.500	0.087	Rank species according to its position in a nestedness matrix and based on the nestedness NODF metric.	[97]
Betweenness (WB)	Weighted	0.014	0.000	Proportion of the shortest paths through node *A*. Centrality of *A* in the network by its position between other nodes.	[98], for more references check the *bipartite* manual for R
Closeness (WC)	Weighted	0.014	0.000	Centrality of *A* in the network by its path lengths to other nodes. Calculated as the inverse of the average distance from *A* to all other nodes.	[98], for more references check the *bipartite* manual for R
Species Strength (SS)	Weighted	1.123	2.030	Quantifies the species' relevance across all its partners as the sum of all its dependencies.	[99–100]
Effective Partner (EP)	Weighted	2.149	1.186	Effective number of partners.	[101]
Species Specificity (SSP)	Weighted	0.211	0.022	Coefficient of variation of interactions.	[102]
Standardized Specialization (*d*)	Weighted	0.757	0.067	Quantifies how specialized is *A* in relation to the partners of the other level that are on offer.	[103]
Corrected Specialization (*d'*)	Weighted	0.839	0.054	*d* value re-arranged between the max value and the heuristically-founded min value.	[103]

To measured cumulative secondary extinctions (*Bipartite*, R) at the plant and frugivore trophic level, we replicated the matrix and from each manually removed one-plant nodes with higher connectance and betweenness indexes values. We subsequently estimated robustness of each matrix using *Bipartite* and randomly removing species from the matrix. Robustness, the area beneath the secondary extinction slope, estimates the resistance of the network to extinction.

Finally, we compared all these network indices with an approach of energy flow through ecosystem levels, which estimates the amount of dry pulp biomass going from each plant to the herbivore level. This estimate was based on the rate of consumption for each species (fruits.h^-1^.tree^-1^), proportion of pulp in the fruit, crop duration (mo), and adult plant density (trees.ha^-1^). All information from plant traits and plant demography can be found in [51, 53]. We also compared with a list of potential keystone species in the area based on its production on scarcity periods, temporal consistency and the number of consumers [43].

## Results

**Fig 1:**
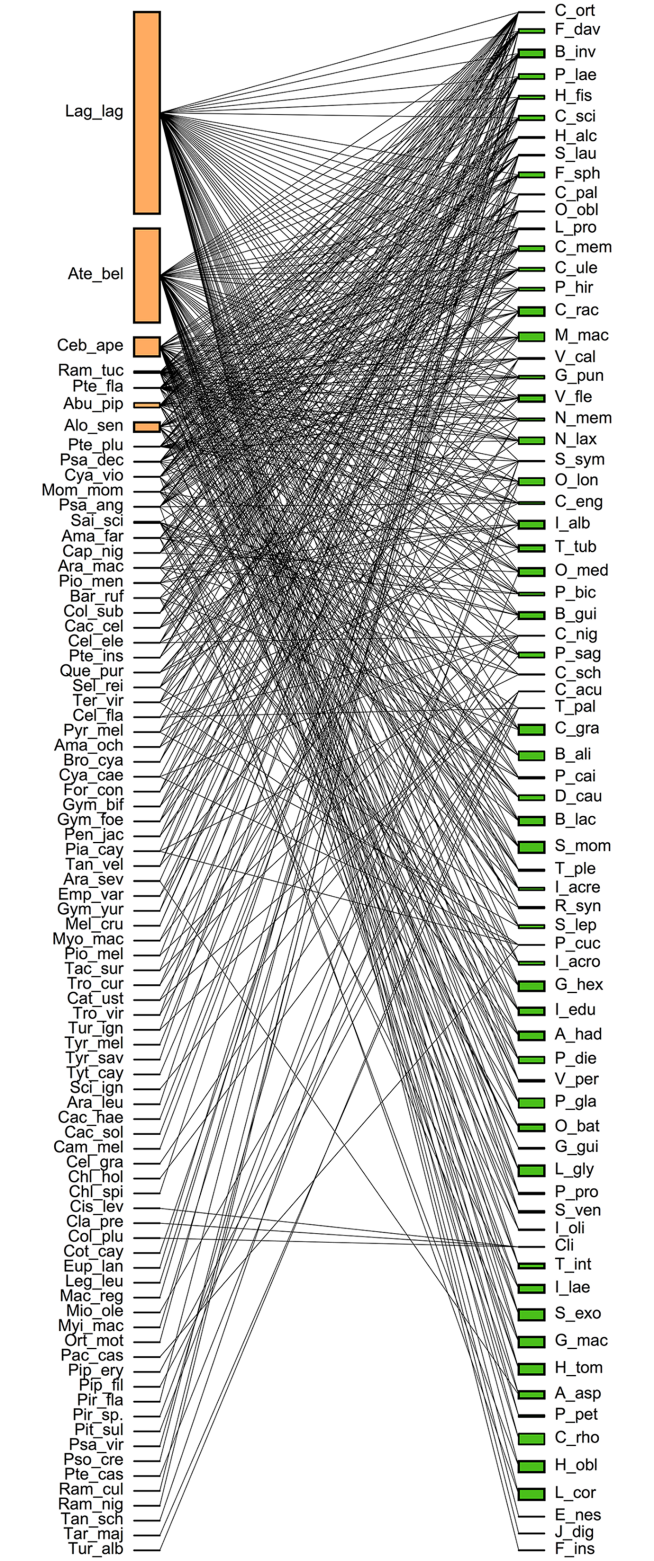
Frugivore-plant network for the species studied in Tinigua National Park, Colombia. The most connected frugivores were woolly monkeys (*Lagothrix lagothricha*), spider monkeys (*Ateles belzebuth*), brown capuchins (*Cebus* or *Sapajus apella*), white-throated toucan (*Ramphastus tucanus*), ivory-billed toucan (*Pteroglossus flavirostris*), common piping-guan (*Pipile pipile*) and howler monkeys (*Alouatta seniculus*). The complete list of plant species appears in Table 1; animal species names are provided in the Supplementary Material (Tables C and SD).

Overall, the number of frugivore species varied widely among plants, being *Jacaratia digitata* (Caricaceae) the one with the lowest number of visitors (two species) and *Coussapoa orthoneura* the highest (58 species, Table 2). Primates dominated this habitat-wide canopy frugivore assemblage, being responsible for 64% of the fruits manipulated across species, while birds removed less than one third of fruits (32%). Woolly monkeys (*Lagothrix lagothricha*) were responsible for manipulating approximately 35% of the fruits, and were the most abundant frugivores in the area reaching 41–50 individuals.km^-2^. Spider monkeys (*Ateles belzebuth*) scored second in terms of the percentage of fruit manipulation across all plant species (19%). These two primate species were observed visiting fruit trees frequently and used a large and diverse set plant species (Fig 1). Overall, 79% of the plants showed a positive association between frugivore abundance and visitation time (Table 2). On average, frugivore density explained 40% of the variation in visit time, and up to 94% of the variation in some tree species.

**Table 2:** List of plant species included in analyses, showing the sample size (hours of observations), the total number of frugivores known to consume the fruits, the proportion of fruits handled by primates, seed predatory birds (e.g. Psittacidae) and other birds, and the coefficient of determination (R^2^) indicating the proportion of the variation explained by the abundance of frugivores.

Species	Sample size (h)	Known consumers	Monkeys	Predatory Birds	Other birds	R^2^ explained by abundance
*Alibertia cf*. *hadrantha*	39	5	82	0	18	**0.94**
*Pourouma bicolor*	70	8	95	5	0	**0.88**
*Crepidospermum rhoifolium*	35	4	100	0	0	**0.87**
*Brosimum guianensis*	31	11	96	2	1	**0.85**
*Garcinia macrophylla*	101	3	97	3	0	**0.84**
*Gustavia hexapetala*	94	4	98	2	0	**0.83**
*Leonia glycycarpa*	39	3	100	0	0	**0.82**
*Spondias mombin*	95	5	100	0	0	**0.82**
*Helicostylis tomentosa*	36	5	100	0	0	**0.81**
*Pouteria procera*	33	4	100	0	0	**0.80**
*Socratea exorrhiza*	30	4	99	0	0	**0.79**
*Spondias venulosa*	28	3	100	0	0	**0.78**
*Maytenus cf*. *macrocarpa*	23	13	80	0	20	**0.77**
*Talisia intermedia*	38	3	100	0	0	**0.75**
*Hymenaea oblongifolia*	114	5	99	0	1	**0.75**
*Laetia corymbulosa*	36	3	99	0	1	**0.75**
*Protium sagotianum*	50	6	90	10	0	**0.71**
*Inga acreana*	50	6	100	0	0	**0.70**
*Jacaratia digitata*	40	2	100	0	0	**0.69**
*Eugenia nesiotica*	30	4	100	0	0	**0.68**
*Brosimum lactescens*	37	10	100	0	1	**0.66**
*Cayaponia granatensis*	32	6	95	0	6	**0.64**
*Ficus insipida*	34	5	100	0	0	**0.61**
*Neea aff*. *laxa*	33	11	77	0	23	**0.59**
*Inga edulis*	74	6	95	0	5	**0.56**
*Protium glabrescens*	36	6	99	0	1	**0.55**
*Cestrum racemosum*	39	19	82	0	17	**0.53**
*Pourouma petiolulata*	40	4	100	0	0	**0.51**
*Pseudolmedia laevigata*	32	32	68	13	19	**0.49**
*Pseudomalmea diclina*	45	4	100	0	0	**0.49**
*Cecropia membranacea*	109	32	65	0	35	**0.48**
*Trichilia pleeana*	46	6	76	0	24	**0.48**
*Bursera inversa*	101	31	74	0	26	**0.46**
*Inga alba*	41	8	72	8	20	**0.45**
*Inga acrocephala*	38	4	83	0	17	**0.39**
*Oxandra mediocris*	36	9	66	0	34	**0.38**
*Apeiba aspera*	115	3	64	37	0	**0.33**
*Cecropia sciadophylla*	59	21	72	0	28	**0.29**
*Trichilia tuberculata*	55	15	74	0	26	**0.29**
*Henriettella fissanthera*	98	31	32	0	68	**0.28**
*Ficus davidsonii*	38	36	51	0	49	**0.28**
*Pseudolmedia hirsuta*	39	15	88	10	2	**0.25**
*Brosimum alicastrum*	36	7	81	0	19	**0.25**
*Celtis schippii*	38	15	91	0	9	**0.25**
*Castilla ulei*	40	12	73	0	27	**0.24**
*Oenocarpus bataua*	81	6	88	12	0	**0.21**
*Ficus sphenophylla*	47	43	49	0	51	**0.21**
*Dendropanax caucanus*	46	6	52	0	48	**0.19**
*Swartzia aff*. *leptopetala*	30	5	91	0	9	**0.19**
*Guatteria cf*. *punctata*	35	10	38	0	62	**0.17**
*Virola peruviana*	45	4	86	0	14	**0.16**
*Sapium laurifolium*	25	25	23	0	77	**0.16**
*Inga cf*. *olivacea*	31	4	100	0	0	**0.15**
*Virola flexuosa*	69	15	60	0	40	**0.13**
*Iryanthera laevis*	23	3	79	0	21	**0.12**
*Ocotea longifolia*	34	18	74	0	26	**0.11**
*Cecropia engleriana*	76	11	17	0	83	**0.08**
*Hieronyma alchorneoides*	30	40	20	2	78	0.02
*Virola calophylla*	33	15	6	0	94	0.02
*Pouteria caimito*	22	5	40	0	60	0.02
*Guarea guidonia*	45	3	0	0	100	0.01
*Laetia procera*	44	20	10	0	90	0.01
*Rhodostemonodaphne synandra*	31	5	2	0	98	0.01
*Nectandra membranacea*	39	11	12	0	88	0.01
*Clusia palmicida*	32	21	2	0	98	0.01
*Coussapoa orthoneura*	32	58	1	2	97	0.00
*Psittacanthus cucullaris*	34	7	0	0	100	0.00
*Ocotea oblonga*	35	17	1	0	99	0.00
*Souroubea sympetala*	31	23	4	0	96	0.00
*Clusia nigrolineata*	31	9	0	0	100	0.00
*Trichilia pallida*	33	7	0	0	100	0.00
*Casearia aculeata*	30	9	0	0	100	0.00

**Fig 2:**
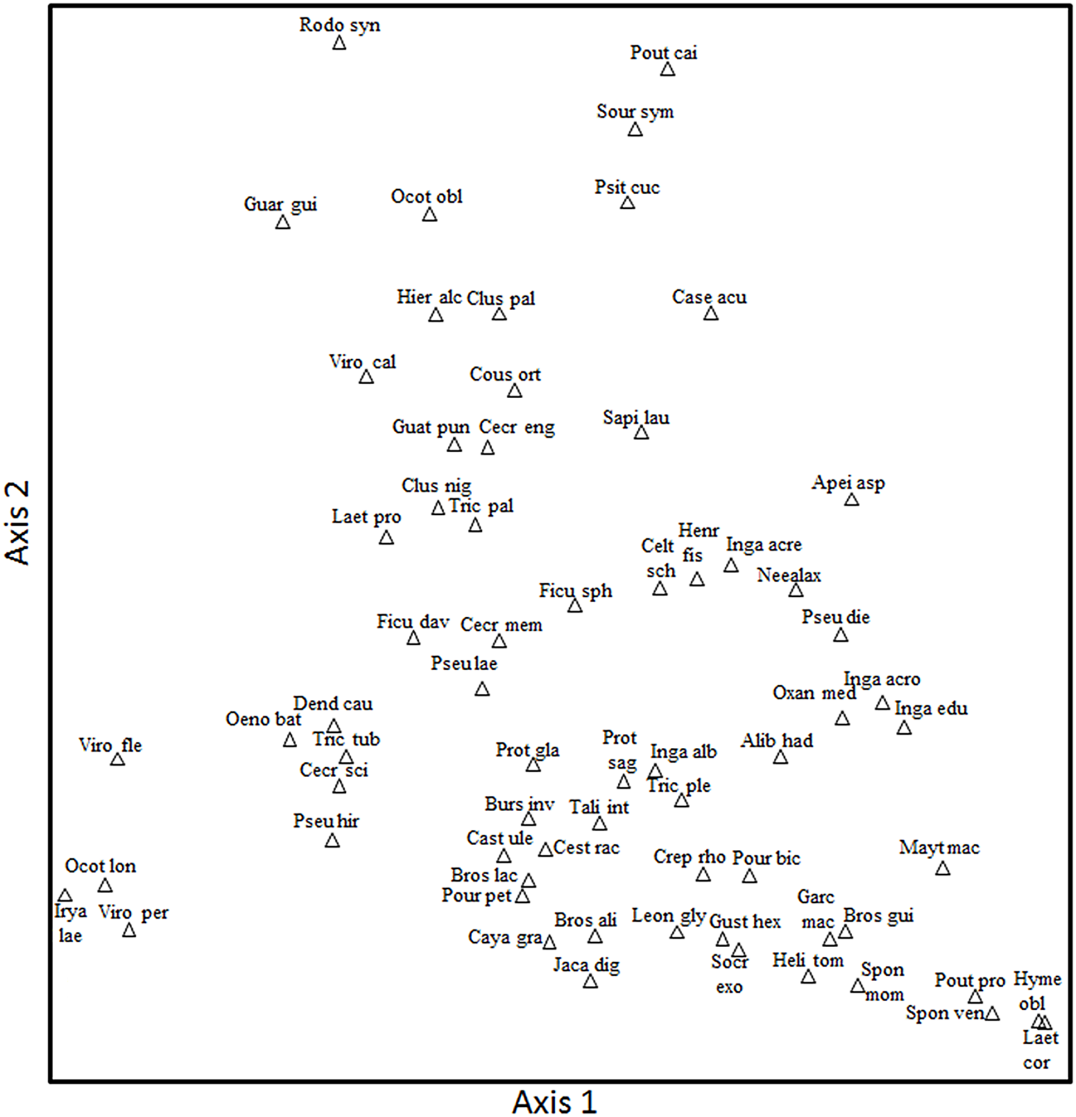
Principal components analysis based on the number of fruits consumed by different frugivore species of 73 canopy plants in an upper Amazonian forest.

The ordination of plants based on frugivore visits did not show discrete groupings, but showed a large effect from the two most important frugivore primates. The first axis of the ordination showed a strong distinction in the visitation rates of *Lagothrix* (r = 0.83), the predominant consumer for the species located to the right and *Ateles* (r = -0.60) to the left (Fig 2). The second axis showed low values for plants consumed by primates and large values for plants consumed mainly by birds (e.g. *Pipile pipile*: r = 0.48). A canonical analysis showed that in terms of fruit traits, lipid content and fruit protection were the most important variables associated with the consumption of plant species, where *Ateles* and several other birds (e.g. *Capito niger*, *Catharus ustulatus*, *Cyanocorax violaceus*, *Empidonomus varius*, *Gymnoderus foetidus*, *Pipile pipile*,) tended to ingest fruits with high lipid content. Fruit protection was associated to high consumption by *Lagothrix*.

### Generalized vs. specialized dispersal systems

**Fig 3:**
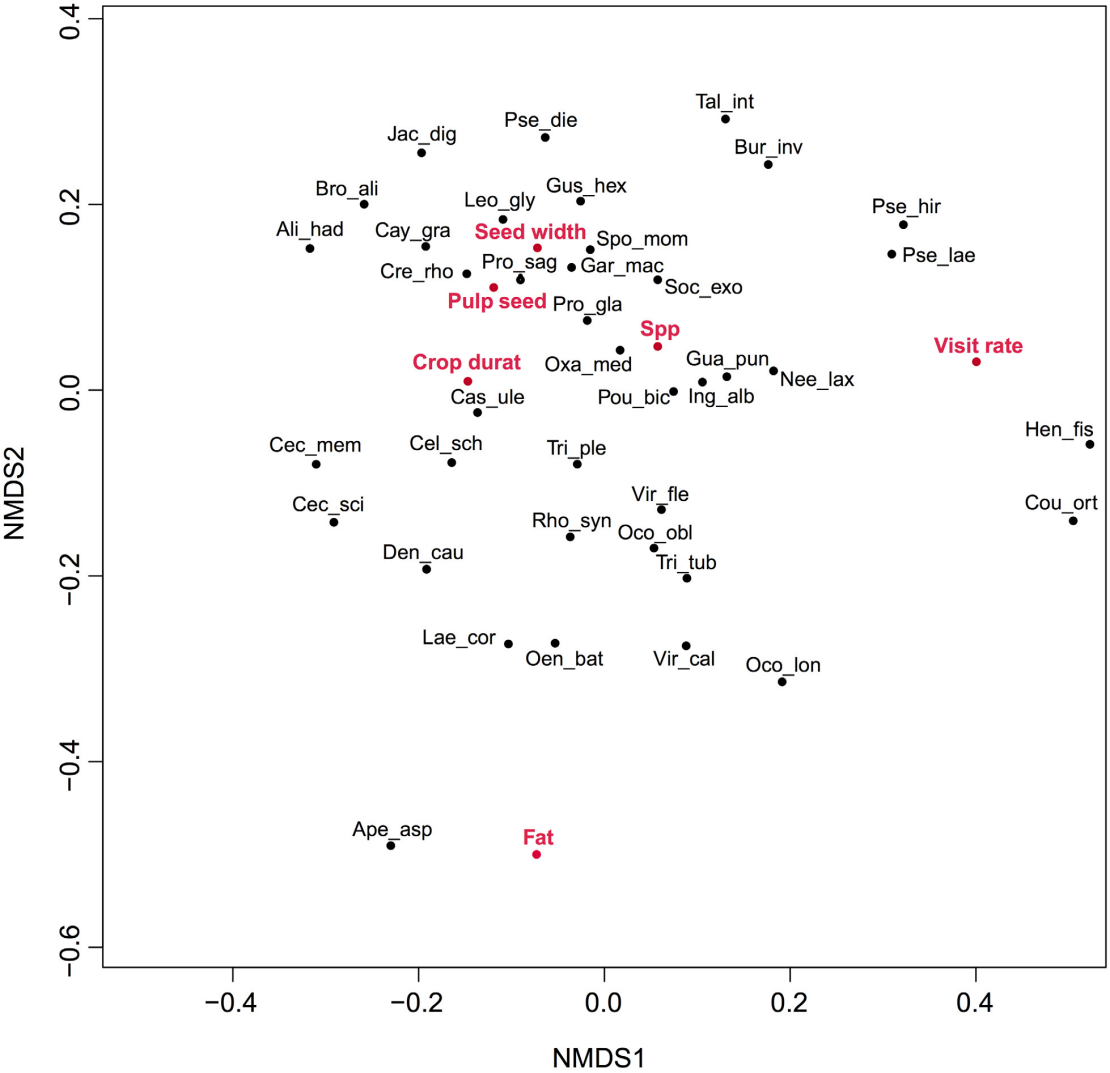
NMDS ordination of plant species based on plant traits that have been used to define specialized vs. generalized dispersal systems. The relevance of each trait is also indicated, for instance, showing that the x-axis is positively associated with visitation rate and that the y-axis is negatively associated with the concentration of lipids in fruit pulp.

We found no support for the expectations of specialized and generalized dispersal systems since none of seven predictions based on our hypothesis were supported (Table 3). In addition, the ordination did not adjust to two groups (Fig 3), but a relatively homogeneous distribution on points, suggesting that most species are visited by the same frugivores. The most distinguishable species (*Henrietella fissanthera* and *Coussapoa orthonerua*) are located to the right of the ordination, and are characterized by high visit rates, very small seeds and large crops. However, other small seeded species are at the opposite left side (such as *Apeiba aspera*, *Cecropia* spp. and *Laetia corymbulosa*). This *Laetia* species is just close to *Oenocarpus bataua*, a large seeded plant. Therefore, neither pulp quality or fruit presentation seem to group generalized and specialized plants. Finally, the degree of specialization (*d’*) did not show a bimodal distribution for plants and animals (Fig A in S1 Appendix).

**Table 3:** Association between nutrient content in fruit pulp (% lipids) and other plant-frugivore traits. The table shows the expected relationships according to the generalized vs. specialized hypothesis, and the coefficients of determination.

Traits	Expected	R^2^
1) % lipids vs. Crop duration	+	0.03 ns
2) % lipids vs. Seed size	+	0.01 ns
3) % lipids vs. Frugivore size	+	0.06 ns
4) % lipids vs. Crop size	-	0.01 ns
5) % lipids vs. Visitation rate	-	0.01 ns
6) % lipids vs. Richness	-	0.09 ns
7) % lipids vs. Regeneration below parents	-	0.01 ns

### Phylogenetic ensemble and potential filters

**Fig 4:**
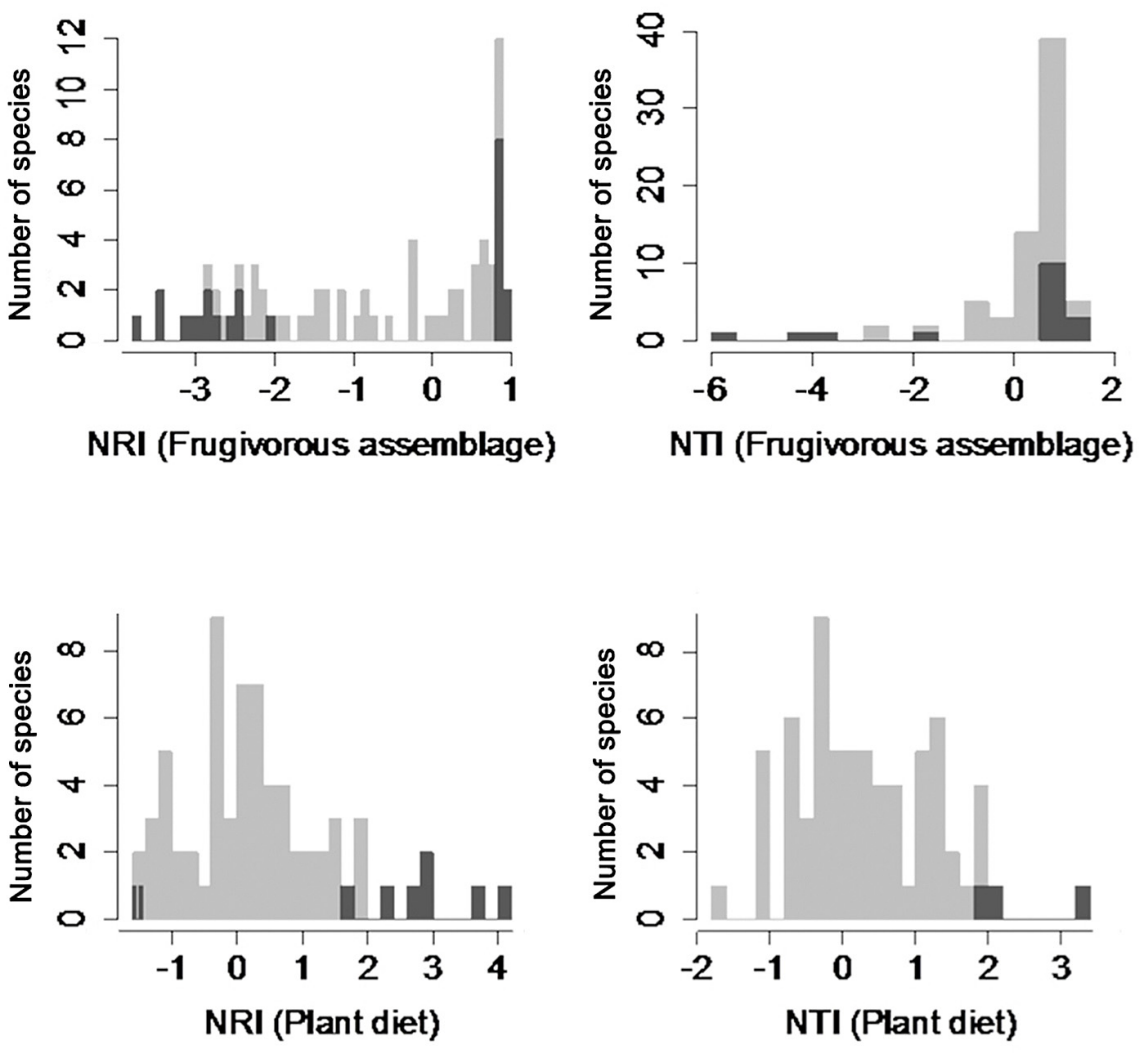
Frequency of species showing aggregated, random and dispersed patterns of phylogenetic aggregation according to NRI and NTI indices. Black bars represent species that differ from random (i.e. are more aggregated or dispersed than expected by chance, gray bars).

We did not find strong evidence for the existence of effective filters, restricting the consumption of particular taxonomic groups and most plant species showed phylogenetic ensembles undistinguishable from random (Fig 4). For example, according to the NRI, 67% of the plant species did not show any particular pattern on taxonomic ensemble, 15% showed grouping patterns because frugivory was biases towards primate species (Table A in S1 Appendix). Out of these 11 species, four had protective husks (*sensu* [21]): *Leonia glycycarpa*, *Pourouma petiolulata*, *Pouteria procera*, and *Talisia intermedia*), six have unprotected fruits (*Brosimum lactescens*, *Eugenia nesiotica*, *Helicostylis tometosa*, *Jacaratia digitata*, *Socratea exorrhiza*, and *Spondias venulosa*) and, and one species has dehiscent capsules (*Crepidosmpermum rhoifolium*). Only two species showed aggregated patterns (3%), due to the preponderance of bird consumption (*Casearia aculeata* and *Psittacanthus cucullaris*). Four species showed over-dispersed patterns of aggregation according to NRI (*Ficus davidsioni*, *F*. *sphenophylla*, *Guatteria* cf. *punctata*, and *Ocotea longifolia*), and were frequently ingested by both birds and monkeys.

According to the NTI, 75% of the plant species did not show any particular pattern on taxonomic ensemble, 18% showed grouping patterns because frugivory was biases towards primate species. In addition to the species mentioned, six species showed phylogenetic aggregation (*Cayaponia granatensis*, *Clusia nigrolineata*, *Inga alba*, *Laetia procera Spondias mombin*, and *Virola flexuosa*). Only *Nectandra membranacea* and *Ocotea longifolia* showed overdispersed patterns according to the NTI index (Table B in S1 Appendix).

Frugivore species were observed to feed, in general, on a variety of species of different taxonomic groups, and most frugivores showed random patterns of taxonomic preference (Tables C and D in S1 Appendix). The only frugivore showing consistent patterns of phylogenetic clustering (looking at both indices) was the howler monkey, which relies heavily on species of the Moraceae family.

### Keystone plants

Based on the pulp biomass going to the frugivore level, the highest-ranking plants were three abundant *Pseudolmedia* species (*P*. *laevis*, *P*. *hirsuta* and *P*. *laevigata*), *Spondias* spp., *Oxandra mediocris*, *Crepidospermum rhoifolium*, and *Talisia intermedia*. The list of plant species potentially acting as keystones for frugivores (based on their high and consistent productivity during periods of fruit scarcity, and their consumption by a variety of frugivores [43]) shared only four species with the list of 20 most consumed species in terms of biomass (Table 4).

**Table 4:** Most important plant species for the frugivore community in Tinigua National Park, according to two approaches. Left side: Pulp biomass going to the frugivore level (taking into account plant density of reproductive trees, crop size, pulp in the fruit, and the amount of fruits consumed). Right side. Plant species producing significant amounts of fruits in periods of scarcity for a variety of consumers (see details in [43]). The plant species in common in both lists are highlighted in bold.

Species	Biomass of pulp consumed/ha.day	Species	Keystone Index
*Pseudolmedia laevis*	16938	***Oenocarpus bataua***	7.32
***Pseudolmedia hirsuta***	11942	***Cecropia membranacea***	6.36
*Pseudolmedia laevigata*	8709	*Bursera inversa*	5.90
*Spondias venulosa*	3848	*Ficus davidsoni*	5.41
*Oxandra mediocris*	2660	***Gustavia hexapetala***	4.80
*Crepidospermum rhoifolium*	2578	*Brosimum alicastrum*	4.51
*Talisia intermedia*	2182	*Ficus sphenophylla*	4.48
*Socratea exorrhiza*	1999	*Brosimum guianense*	4.31
*Alibertia cf*. *hadrantha*	1705	*Iriartea deltoidea*	4.26
***Oenocarpus bataua***	1674	*Astrocaryum chambira*	3.98
*Protium glabrescens*	1208	*Apeiba aspera*	3.79
*Inga acreana*	1174	***Pseudolmedia hirsuta***	3.77
*Garcinia macrophylla*	997	*Brosimum utile*	3.77
***Gustavia hexapetala***	966	*Ficus americana*	3.72
*Protium sagotianum*	938	*Dialium guianense*	3.58
*Coussapoa orthoneura*	920	*Henriettella fissanthera*	3.28
*Spondias mombin*	891	*Ficus trigonata*	3.16
*Castilla ulei*	748	*Pourouma bicolor*	3.07
***Cecropia membranacea***	652	*Enterolobium schomburgkii*	2.72
*Brosimum lactescens*	586	*Ficus trigona*	2.53

**Fig 5:**
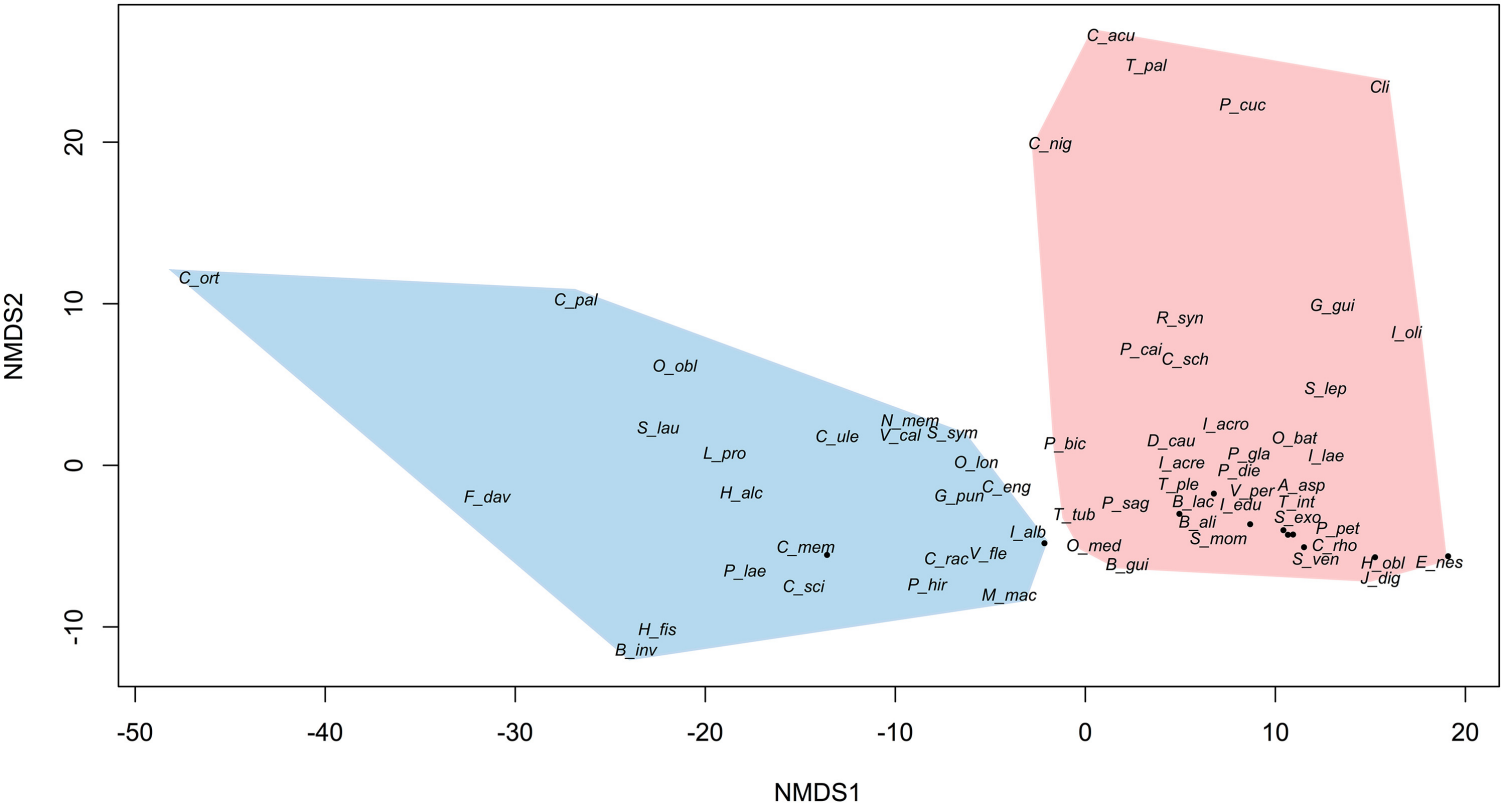
NMDS ordination of plant species consumed by frugivores, based on the indices describing the structure of frugivore-plant network. Blue represents core species while green represents periphery species based on the classification used by [69].

Mean values and variance for the measured indices for the plants’ trophic level are summarized in Table 1. The total NODF of the matrix (30.82) revealed evidence of nestedness in the community of frugivores and that such nestedness was significantly different than expected under null models (NODF_ER_ = 8.83 STD = 0.491; NODF_CE_ = 14.46 STD = 0.911, both p = 0). Both, highly and less connected frugivores used mainly highly connected plants and there was little evidence of compartmentalization due to specialization of plant consumption. The observed H2’ for the matrix was 0.536, while the distribution of H2’ obtained for the null matrices had a mean of 0.0098 and a standard deviation of 0.00087. The intermediate H2’ value indicates that the matrix was not as specialist as it would be expected given the structure of the network. This is congruent with the estimated nestedness of the network and the distribution of *d’* for plants and frugivores (Fig A in S1 Appendix). The NMDS scaling summarized variables related to degree and nested ranking in the first axis and variables related to betweenness and closeness in the second axis (Fig 5). Axis 1 showed *Bursera inversa*, *Clusia palmicida*, *Coussapoa orhtoneura*, *Ficus davidsionii*, and *Sapium laurifolium* as the most relevant species in terms of degree and nested ranking. Axis 2 showed *Bursera inversa*, *Cecropia sciadophylla*, *Henriettella fissanthera*, *Jacaratia digitata*, *Maytenus macrocarpa*, and *Pseudolmedia hirsuta* as the most relevant species in terms of betweenness and closeness. Additionally, both axes highlighted *Bursera inversa*, *Cecropia membranacea*, *C*. *sciadophylla*, *Cestrum racemosum*, *Ficus sphenophylla*, *Henriettella fissanthera*, *Pseudolmedia hirsuta*, and *P*. *laevigata* as relevant species. Less connected, central nodes seem to aggregate toward positive values in both axes. *Crepidospermum rhoifolium*, *Oxandra mediocris*, *Pseudolmedia hirsuta*, *P*. *laevigata*, and *Spondias venulosa* were consistently related with high connectance.

The correlation analyses revealed strong associations among nine of the 11 indices used in the network analysis (Table 5). As expected, correlations depend on their main structural principle, so that indices based on the sum of total interactions per species (D, ND, NR, and G), centrality and specialization (*d*, *d’*, and WC), and the quantitative variation of interactions (EP and SSP) highly correlate within groups. From the total of plant species evaluated, 20 were highlighted for six or more indices as nodes highly and strongly connected (Table E in S1 Appendix). From these, *Bursera inversa*, *Cecropia membranacea*, *Cestrum racemosum*, *Coussapoa orthoneura*, *Laetia procera*, *Ocotea oblonga*, and *Pseudolmedia hirsuta*, were the most relevant species in terms of pulp biomass entering the frugivore level. However, none of the network indices were even slightly correlated with the amount of pulp biomass going to the frugivore level (column 1 in Table 5).

**Table 5:** Comparison of methods to estimate keystone plants from the amount of pulp biomass (Pulp) going to the frugivore level and different indices using topological networks (described in Table 1), based on correlations. High correlations (r > 0.6) are highlighted.

	Pulp	ND	WB	WC	*d'*	*d*	NR	D	SS	EP	SSP
Normalized Degree (ND)	0.10										
Betweenness (WB)	-0.03	0.55									
Closeness (WC)	0.13	0.24	0.20								
Corrected Specialization (*d'*)*	0.15	-0.07	0.07	**0.72**							
Standardized Specialization (*d*)*	0.15	-0.02	0.09	**0.80**	**0.96**						
Nested Rank (NR)*	0.10	**0.93**	0.53	0.30	0.02	0.06					
Degree (D)	0.10	**1.00**	0.55	0.24	-0.06	-0.01	**0.93**				
Species Strength (SS)	-0.09	**0.67**	0.47	-0.30	-0.50	-0.52	0.54	**0.67**			
Effective Partner (EP)	0.07	0.51	0.15	0.00	-0.18	-0.22	0.42	0.51	0.44		
Species Specificity (SSP)*	0.06	0.34	0.06	-0.06	-0.12	-0.17	0.31	0.34	0.24	**0.89**	
Generality (G)	0.10	**1.00**	0.55	0.24	-0.06	-0.01	**0.93**	1.00	**0.67**	0.51	0.34

*Inversed values as described in the methods.

The plant species described above where subtracted one by one from the network to evaluate the network robustness after the secondary losses in both, plants and frugivores. Robustness values for the unaltered network reveals asymmetries between plant and frugivore levels (R = 0.83 and R = 0.68, respectively). After individually removing plant species with high values of connectance and betweenness, robustness values for the plant trophic level for all networks fall near R = 0.8 (mean R = 0.82, var = 0.0002). However robustness to frugivore extinction did not vary significatively among networks after removing one by one plant species (mean R = 0.67, var = 0.0001), and it was lower for the frugivores’ trophic level, suggesting stronger repercussions over frugivores (i.e. more frugivore species than plant species will be lost after removing plant species). Variation in robustness among networks must be interpreted carefully since qualitative analysis might not be sensible enough [70].

## Discussion

Our results highlight a plant-animal sub-system (including just diurnal canopy frugivores) where the majority of fruits are manipulated by the most common frugivores in the area, and dominated by two fruit ripe specialists (*Lagothrix lagothricha* and *Ateles belzebuth*) [71]. The importance of monkeys in frugivory and seed dispersal has been emphasized in different reviews [72–75]. Studies including different taxonomic groups of frugivores at the community level in the tropical forest suggest a high relevance of birds, followed by mammals [76–78]. Primates dominated in our system of diurnal frugivory, and this was not expected because seed dispersal by birds has been broadly documented and popularized [79]. We think that this bias may be caused in part, because birds are more widely distributed than primates, and large primates are more vulnerable than birds to habitat destruction and hunting [80], making birds common components in many places. However, most studies evaluating the role of primates as effective seed dispersers have highlighted positive effects [71–74, 81], and at least one comparative study in Madagascar emphasized more important roles of primates than other frugivores [75]. Perhaps, these primate-dominated communities are more frequent in undisturbed tropical forests than currently acknowledged.

The inclusion of understory plants will probably decrease the effect of large atelines, because they do not feed on fruits near the ground and that our estimates may be slightly biased for some frugivores. For instance, *Cassearia aculeata*, the smallest species included in the study, was consumed only by bird species. In addition, it is clear that we are underestimating the role of some frugivores, such as bats and terrestrial animals because here we did not include nocturnal observations and terrestrial frugivores may not approach fruiting trees in the presence of researchers. For example, for some *Cecropia* and green-syconium fig species, the role of bats seems to be important [82–83].

The fact that primates dominate our plant-frugivore system seems to be tightly associated with the high density of monkeys in the study area. In particular, the density of ateline monkeys has been estimated as 68.7 individuals.km^-2^, comparable only to pristine forest on relatively rich western Amazonian soils, such as Cocha Cashu Biological Station (57.4), Tiputini Biological Station (42.5), El Trueno Experimental Ranch (37.9) (references in [84]). In poor soils, ateline density tends to decrease (even in non-hunted areas), such as Urucu (11.7) or Caparu (15.0) [85], but we do not anticipate a reason why monkeys would not dominate these plant-animal systems. In principle, bird abundance may also be reduced in poor soils and monkeys are dominant or codominant within the canopy dwellers [86]. Actually primates are dominant over avian species and our observations show that only tayras (in very few occasions) may rival access to canopy fruits and woolly monkeys dominate among primates [52]. Overall, our results show that plant-animal relationships are highly explained by the abundance of frugivores, plant filters have allowed the use of fruit by different taxa, and since monkeys show priority access to fruiting trees, their density is the most critical factor explaining the frugivore assemblage.

The most frugivorous primates, woolly monkeys and spider monkeys, show the highest fruit-diet overlap in this community [52] and preferentially consume productive plant species. Their dissimilarity in fruit diet was the main determinant in the ordination of plant species (based on consumer frequency). It has been noted that woolly monkeys prefer common plants, with large crops, and sugar rich pulp (e.g. *Brosimum* spp., *Cecropia membranacea*, *Gustavia hexapetala*, *Henriettella fissanthera*, *Inga* spp., *Pourouma bicolor*, and *Spondias mombin*); but avoid other common plants, characterized by high lipid content and the presence of secondary compounds [53]. On the other hand, spider monkeys rely on *Cecropia membranacea*, *Gustavia hexapetala*, *Oenocarpus batua*, *Trichilia tuberculata*, and *Virola flexuosa* [52]. Therefore, both monkeys share many species, but differ in others, the case of *O*. *bataua* being the most striking (a palm that is not ingested by woolly monkeys). Perhaps, the use of some lipid rich and astringent fruits by spider monkeys is related to their use of salt licks [87], which are not used by woolly monkeys. We hypothesize that the incorporation of fine-grained analyses on pulp secondary compounds will play a significant role at explaining part of the variation in frugivore selection.

Our results do not show support for the existence of generalized vs. specialized dispersal systems [88]. The tropical plants included in our studies do not have the associations predicted by this paradigm, and none of the expected correlations were supported. Therefore, the evolution of plant traits such as pulp composition, crop size, crop duration and their capacity to recruit below parents seems to have originated in different plant species without the necessary tandem evolution of correlated characters. Our results agree with a number of studies finding no support for the existence of these generalized vs. specialized systems [27, 81, 89] and suggest that tropical forest are no exception.

For the majority of plants, we did not detect strong filters making plants able to restrict the use of fruits by taxonomic groups. Therefore, only a small proportion of the species showed aggregated ensemble patterns and the cases in which we found aggregation (mainly because the plants were visited just by birds, or just by primates) suggest at least three potential factors. First, fruit protection by husks seems to restrict the use of fruit by primates [21], mediated by their handling abilities. Interestingly, Psittacidae (parrots and allies) that show enhanced abilities, have also shared use with primates in some protected species (i.e. *Apeiba aspera*, *Pouteria caimito*). Second, forest strata and plant height may influence the frugivore ensemble (as previously found [30]), in part because small understory plants may not support large primates. Third, although we lack analyses of secondary compounds for most of the studied species, we think that there is a strong potential of chemicals as frugivore filters. For instance, five out of seven of the unprotected species consumed mostly by primates have latex or odoriferous exudates that suggest the presence of particular chemicals.

No consensus exists among researches about the validity and relevance of certain network indices used to predict keystone species [44]. In addition to the structural properties of one node, it is necessary to evaluate the effectiveness of energy transmission through this node, using different weighted indices. The fact that network indices were not correlated with the amount of pulp biomass going to frugivores is probably caused by the fact that plant density varies between species. Then, the most important plants according to this estimate are common trees, usually with large crops that attract frugivores even in periods of fruit abundance (e.g. *Pseudolmedia* spp.). For this reason, the most important plants for frugivores in terms of pulp biomass do not necessarily correspond to species producing fruits in scarcity periods [40], and experimental tests seem necessary to understand the effects on frugivores after the removal of plants that produce fruit in scarcity and abundance periods. For us, intuitively, it makes sense that for assemblages dominated by large primates that may store fat in periods of fruit abundance, the overall transfer of pulp biomass is a good measure of the importance of plants for frugivores. Interestingly, none of the network indices were correlated with our approach of energy flow, suggesting that a fundamental part of the interaction is missing (even for weighted indices). We propose that estimates of biomass flow, taking into account the abundance of individuals, are required to better understand the relevance of plant species on frugivorous guilds and we think that the use of these methods for conservation practice may be misleading.

Overall, we have found a preponderance of generalist interactions, where the abundance of frugivores is a key factor explaining the composition of fruit consumers [90]. On the other hand, several studies using primates have found that their abundance is highly determined by the productivity of their main fruit resources [91–92]. This leads to questions of causal relationships and which state comes first, since legitimate frugivores may also affect the composition of plant communities [93, 94, 84]. It is still possible that in these systems, when the abundance of frugivores increases because of elevated resource production and some efficient seed dispersers may increase the density of the plants supporting frugivores, then both mutualists may reach high densities [95]. Of course, in complex systems such as tropical forests, many factors are usually playing significant roles and there is also a large chance to cheat in mutualistic systems [96]. However, in our study system, it seems that abundant primates are playing efficient seed dispersal roles that may affect the regeneration patterns of the forest [84, 94]. Perhaps, there is no need to know what process comes first, and the evidence suggests that both nutritional demands and seed dispersal are both causal explanations.

## Supporting Information

S1 Appendix Fig A. Distribution of *d*’ values for plants and frugivores. Tables A & B. Patterns of phylogenetic ensemble for the plant species. Tables C & D. Patterns of phylogenetic ensemble for the frugivore species. Table E. Most important keystones according to different network indices.
(DOCX)

S1 Dataset Matrix of the plants studied and the frugivores visiting each plant, in terms of the percentage of fruits handled. (XLSX)
