# Supplementary material for: Frugivory in Canopy Plants in a Western Amazonian Forest: Dispersal Systems, Phylogenetic Ensembles and Keystone Plants
Source: PLoS One. 2015 Oct 22;10(10):e0140751. doi: 10.1371/journal.pone.0140751 (PMC4619584; doi:10.1371/journal.pone.0140751)
Supplement: S1 Appendix — Fig A. Distribution of d’ values for plants and frugivores. Tables A & B. Patterns of phylogenetic ensemble for the plant species. Tables C & D. Patterns of phylogenetic ensemble for the frugivore species. Table E. Most important keystones according to different network indices. (DOCX) [file pone.0140751.s001.docx]

**S1 Appendix. Figure A**. Distribution of *d’* values for plants and frugivores. Note that the distribution of *d’* for frugivore animals is around intermediate values. For plants, the distribution of *d’* is skewed towards values indicating specialization, however half of the plants showed values of intermediate specialization.

**
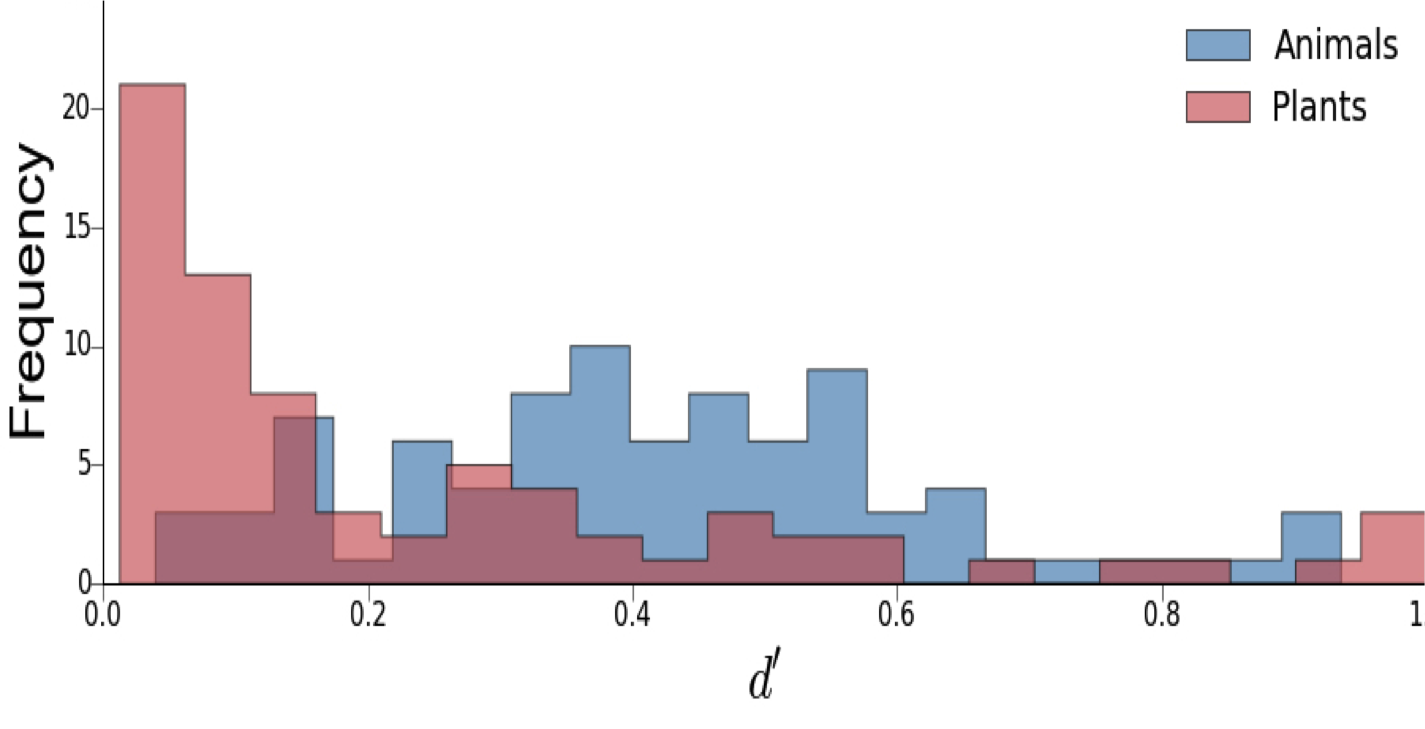
**

**S1 Appendix. Table A**. Patterns of phylogenetic ensemble for the plant species studied, according to the Net Relatedness Index (NRI). Significantly aggregated (positive NRI values) and over-dispersed (negative) patterns are highlighted in the last column.

| Species | N taxa | NRI | P |
| --- | --- | --- | --- |
| *Alibertia cf. hadrantha* | 6 | -1.28 | 0.86 |
| *Apeiba aspera* | 4 | -2.26 | 0.92 |
| *Brosimum alicastrum* | 7 | -1.48 | 0.86 |
| *Brosimum guianensis* | 10 | 0.23 | 0.64 |
| *Brosimum lactescens* | 9 | 0.81 | **0.07** |
| *Bursera inversa* | 26 | -1.73 | 0.90 |
| *Casearia aculeata* | 6 | 0.83 | **0.07** |
| *Castilla ulei* | 11 | -2.29 | 0.95 |
| *Cayaponia granatensis* | 6 | 0.17 | 0.70 |
| *Cecropia engleriana* | 8 | -1.28 | 0.87 |
| *Cecropia membranace* | 21 | -2.64 | 0.98 |
| *Cecropia sciadophylla* | 21 | -2.07 | 0.95 |
| *Celtis schippii* | 11 | -0.32 | 0.88 |
| *Cestrum racemosum* | 16 | -0.86 | 0.89 |
| *Clusia nigrolineata* | 7 | 0.72 | 0.11 |
| *Clusia palmicida* | 17 | 0.79 | 0.17 |
| *Coussapoa orthoneura* | 47 | 0.75 | 0.22 |
| *Crepidospermum rhoifolium* | 3 | 0.83 | **0.06** |
| *Dendropanax caucanus* | 7 | -2.77 | 0.99 |
| *Eugenia nesiotica* | 2 | 0.88 | **0.02** |
| *Ficus davidsionii* | 29 | -3.51 | **1.00** |
| *Ficus insipida* | 3 | 0.66 | 0.12 |
| *Ficus sphenophylla* | 28 | -3.12 | **1.00** |
| *Garcinia macrophylla* | 4 | 0.34 | 0.41 |
| *Guarea guidonia* | 3 | -0.13 | 0.79 |
| *Guatteria cf. punctata* | 10 | -2.78 | **0.99** |
| *Gustavia hexapetala* | 5 | 0.67 | 0.12 |
| *Helicostylis tomentosa* | 3 | 0.88 | **0.02** |
| *Henriettella fissanthera* | 27 | -2.91 | 0.96 |
| *Hyeronima alchorneoides* | 31 | -2.21 | 0.93 |
| *Hymenaea oblongifolia* | 2 | -2.78 | 0.94 |
| *Inga acreana* | 5 | 0.53 | 0.25 |
| *Inga acrocephala* | 4 | -1.27 | 0.86 |
| *Inga alba* | 8 | -1.93 | 0.90 |
| *Inga olivacea* | 3 | 0.55 | 0.24 |
| *Inga edulis* | 4 | -0.07 | 0.78 |
| *Iryanthera laevis* | 3 | -3.21 | **0.97** |
| *Jacaratia digitata* | 2 | 0.88 | **0.02** |
| *Laetia corymbulosa* | 2 | -2.71 | 0.94 |
| *Laetia procera* | 15 | -0.66 | 0.83 |
| *Leonia glycycarpa* | 4 | 0.91 | **0.03** |
| *Maytenus cf. macrocarpa* | 9 | -3.06 | 0.95 |
| *Nectandra membranacea* | 9 | -1.86 | 0.94 |
| *Neea aff. laxa* | 9 | -0.33 | 0.87 |
| *Ocotea longifolia* | 12 | -3.60 | **0.99** |
| *Ocotea oblonga* | 13 | -0.27 | 0.83 |
| *Oenocarpus bataua* | 3 | -0.95 | 0.87 |
| *Oxandra mediocris* | 7 | -2.83 | 0.94 |
| *Pourouma bicolor* | 7 | 0.20 | 0.67 |
| *Pourouma petiolulata* | 4 | 0.89 | **0.04** |
| *Pouteria caimito* | 5 | -3.15 | 0.97 |
| *Pouteria procera* | 3 | 0.85 | **0.03** |
| *Protium glabrescens* | 7 | 0.64 | 0.18 |
| *Protium sagotianum* | 7 | 0.80 | 0.11 |
| *Pseudolmedia hirsuta* | 11 | -0.85 | 0.84 |
| *Pseudolmedia laevigata* | 18 | -2.54 | 0.95 |
| *Pseudomalmea dielsiana* | 4 | 0.55 | 0.23 |
| *Psittacanthus cucullaris* | 4 | 0.84 | **0.04** |
| *Rhodostemonodaphne synandra* | 6 | -0.50 | 0.89 |
| *Sapium laurifolium* | 17 | -2.61 | 0.99 |
| *Socratea exorrhiza* | 4 | 0.84 | **0.04** |
| *Souroubea sympetala* | 16 | 0.07 | 0.70 |
| *Spondias mombin* | 4 | 0.86 | 0.03 |
| *Spondias venulosa* | 3 | 0.92 | **0.02** |
| *Swartzia aff. leptopetala* | 5 | -1.52 | 0.90 |
| *Talisia intermedia* | 3 | 0.78 | **0.08** |
| *Trichilia pallida* | 5 | 0.32 | 0.51 |
| *Trichilia pleeana* | 7 | -1.64 | 0.87 |
| *Trichilia tuberculata* | 9 | -2.15 | 0.95 |
| *Virola calophylla* | 11 | -1.21 | 0.83 |
| *Virola flexuosa* | 12 | -2.91 | 0.96 |
| *Virola peruviana* | 4 | -2.20 | 0.95 |

**S1 Appendix. Table B**. Patterns of phylogenetic ensemble for the plant species studied, according to the Nearest Taxon Index (NTI). Significantly aggregated (positive NTI values) and over-dispersed (negative) patterns are highlighted in the last column.

| Species | N taxa | NTI | P |
| --- | --- | --- | --- |
| *Alibertia cf. hadrantha* | 6 | 0.71 | 0.19 |
| *Apeiba aspera* | 4 | -2.06 | 0.92 |
| *Brosimum alicastrum* | 7 | 0.78 | 0.09 |
| *Brosimum guianensis* | 10 | 0.66 | 0.10 |
| *Brosimum lactescens* | 9 | 0.82 | 0.07 |
| *Bursera inversa* | 26 | 0.65 | 0.16 |
| *Casearia aculeata* | 6 | 1.14 | **0.02** |
| *Castilla ulei* | 11 | 0.69 | 0.14 |
| *Cayaponia granatensis* | 6 | 0.91 | **0.04** |
| *Cecropia engleriana* | 8 | 0.71 | 0.17 |
| *Cecropia membranace* | 21 | 0.57 | 0.29 |
| *Cecropia sciadophylla* | 21 | 0.41 | 0.33 |
| *Celtis schippii* | 11 | 0.22 | 0.52 |
| *Cestrum racemosum* | 16 | 0.64 | 0.18 |
| *Clusia nigrolineata* | 7 | 1.07 | **0.03** |
| *Clusia palmicida* | 17 | 0.84 | 0.14 |
| *Coussapoa orthoneura* | 47 | 0.46 | 0.33 |
| *Crepidospermum rhoifolium* | 3 | 1.04 | **0.04** |
| *Dendropanax caucanus* | 7 | 0.24 | 0.51 |
| *Eugenia nesiotica* | 2 | 0.86 | **0.02** |
| *Ficus davidsionii* | 29 | 0.46 | 0.32 |
| *Ficus insipida* | 3 | 0.76 | 0.14 |
| *Ficus sphenophylla* | 28 | 0.03 | 0.63 |
| *Garcinia macrophylla* | 4 | 0.70 | 0.14 |
| *Guarea guidonia* | 3 | 0.14 | 0.56 |
| *Guatteria cf. punctata* | 10 | 0.72 | 0.20 |
| *Gustavia hexapetala* | 5 | 0.78 | 0.10 |
| *Helicostylis tomentosa* | 3 | 0.96 | **0.03** |
| *Henriettella fissanthera* | 27 | 0.63 | 0.21 |
| *Hieronyma alchorneoides* | 31 | -0.51 | 0.84 |
| *Hymenaea oblongifolia* | 2 | -2.90 | 0.95 |
| *Inga acreana* | 5 | 0.74 | 0.14 |
| *Inga acrocephala* | 4 | -0.62 | 0.83 |
| *Inga alba* | 8 | 1.05 | **0.02** |
| *Inga olivacea* | 3 | 0.57 | 0.28 |
| *Inga edulis* | 4 | 0.04 | 0.64 |
| *Iryanthera laevis* | 3 | -3.83 | 0.98 |
| *Jacaratia digitata* | 2 | 0.86 | **0.02** |
| *Laetia corymbulosa* | 2 | -2.91 | 0.95 |
| *Laetia procera* | 15 | 1.05 | **0.03** |
| *Leonia glycycarpa* | 4 | 0.99 | **0.03** |
| *Maytenus cf. macrocarpa* | 9 | 0.02 | 0.66 |
| *Nectandra membranacea* | 9 | -1.98 | **0.97** |
| *Neea aff. laxa* | 9 | 0.36 | 0.40 |
| *Ocotea longifolia* | 12 | -4.95 | **1.00** |
| *Ocotea oblonga* | 13 | -0.72 | 0.88 |
| *Oenocarpus bataua* | 3 | -0.42 | 0.84 |
| *Oxandra mediocris* | 7 | 0.24 | 0.51 |
| *Pourouma bicolor* | 7 | 0.82 | 0.08 |
| *Pourouma petiolulata* | 4 | 1.00 | **0.06** |
| *Pouteria caimito* | 5 | -3.49 | 0.95 |
| *Pouteria procera* | 3 | 0.86 | **0.03** |
| *Protium glabrescens* | 7 | 0.84 | 0.09 |
| *Protium sagotianum* | 7 | 0.83 | 0.14 |
| *Pseudolmedia hirsuta* | 11 | 0.75 | 0.08 |
| *Pseudolmedia laevigata* | 18 | 0.39 | 0.40 |
| *Pseudomalmea dielsiana* | 4 | 0.73 | 0.15 |
| *Psittacanthus cucullaris* | 4 | 0.96 | 0.02 |
| *Rhodostemonodaphne synandra* | 6 | -0.77 | 0.90 |
| *Sapium laurifolium* | 17 | 0.00 | 0.64 |
| *Socratea exorrhiza* | 4 | 0.98 | **0.05** |
| *Souroubea sympetala* | 16 | -0.16 | 0.75 |
| *Spondias mombin* | 4 | 0.84 | **0.04** |
| *Spondias venulosa* | 3 | 0.83 | **0.03** |
| *Swartzia aff. leptopetala* | 5 | 0.07 | 0.58 |
| *Talisia intermedia* | 3 | 1.04 | **0.06** |
| *Trichilia pallida* | 5 | 0.03 | 0.59 |
| *Trichilia pleeana* | 7 | 0.73 | 0.16 |
| *Trichilia tuberculata* | 9 | 0.63 | 0.21 |
| *Virola calophylla* | 11 | -0.12 | 0.69 |
| *Virola flexuosa* | 12 | 0.88 | **0.03** |
| *Virola peruviana* | 4 | 0.60 | 0.22 |

**S1 Appendix. Table C**. Patterns of phylogenetic ensemble for the frugivore species observed, according to the Net Relatedness Index (NRI). Significantly aggregated (positive NRI values) and over-dispersed (negative) patterns are highlighted in the last column.

|  | N taxa | NRI | P |
| --- | --- | --- | --- |
| *Lagothrix lagothricha* | 59 | 1.75 | **0.05** |
| *Ateles belzebuth* | 56 | -1.15 | 0.86 |
| *Alouatta seniculus* | 30 | 4.34 | **0.00** |
| *Sapajus apella* | 44 | 1.20 | 0.12 |
| *Saimiri cassiaquirensis* | 11 | 1.15 | 0.12 |
| *Pipile pipile* | 24 | -0.81 | 0.78 |
| *Amazona* farinosa | 9 | 0.84 | 0.20 |
| *Amazona ochrocephala* | 3 | 1.51 | 0.09 |
| *Ara macao* | 7 | 2.91 | **0.01** |
| *Ara severa* | 3 | 0.15 | 0.34 |
| *Baryphthengus ruficapillus* | 9 | -1.61 | 0.98 |
| *Brotogeris cyanoptera* | 4 | 0.61 | 0.20 |
| *Cacicus cela* | 11 | -1.39 | 0.90 |
| *Cacicus solitarius* | 3 | 0.33 | 0.32 |
| *Capito niger* | 20 | -1.04 | 0.85 |
| *Celeus elegans* | 9 | 0.83 | 0.20 |
| *Celeus flavus* | 6 | 0.16 | 0.42 |
| *Celeus grammineus* | 4 | 0.71 | 0.22 |
| *Chlorophanes spiza* | 6 | 0.72 | 0.23 |
| *Patagioena plumbea* | 7 | -1.12 | 0.87 |
| *Cyanerpes caeruleus* | 10 | 0.04 | 0.45 |
| *Cyanocorax violaceus* | 14 | 0.34 | 0.37 |
| *Dacnis cayana* | 6 | 0.22 | 0.39 |
| *Euphonia laniirostris* | 6 | 0.45 | 0.32 |
| *Euphonia minuta* | 4 | 0.16 | 0.39 |
| *Forpus conspicillatus* | 5 | 0.49 | 0.30 |
| *Gymnopithys bicolor* | 3 | -0.41 | 0.65 |
| *Gymnoderus foetidus* | 3 | -1.25 | 0.92 |
| *Gymnostinops guatimozus* | 2 | 2.05 | **0.06** |
| *Hemithraupis flavicollis* | 2 | -0.02 | 0.38 |
| *Lipaugus vociferans* | 2 | -1.06 | 0.88 |
| *Melanerpes cruentatus* | 5 | 1.69 | **0.06** |
| *Momotus momota* | 17 | 0.33 | 0.37 |
| *Penelope jaquacu* | 3 | -0.92 | 0.79 |
| *Piaya cayana* | 5 | 0.76 | 0.21 |
| *Pionites melanocephala* | 2 | 0.06 | 0.39 |
| *Pionus menstruus* | 7 | 1.31 | 0.10 |
| *Pipra coronata* | 3 | 0.65 | 0.22 |
| *Pipra erythrocephala* | 6 | -0.04 | 0.51 |
| *Pipra filicauda* | 2 | -0.31 | 0.60 |
| *Pipra sp.* | 2 | -0.33 | 0.55 |
| *Piranga olivacea* | 2 | -0.31 | 0.51 |
| *Porphyrolaema porphyriolema* | 3 | 0.22 | 0.40 |
| *Psarocolius angustifrons* | 13 | 2.98 | **0.01** |
| *Psarocolius decumanus* | 16 | 2.91 | **0.00** |
| *Pteroglossus castanotis* | 2 | 0.05 | 0.43 |
| *Pteroglossus flavirostris* | 26 | -1.52 | 0.95 |
| *Pteroglossus pluricinctus* | 8 | 1.59 | **0.08** |
| *Pteroglossus inscriptus* | 16 | -0.43 | 0.64 |
| *Pyrrhura melanura* | 5 | 1.90 | **0.05** |
| *Querula purpurata* | 9 | 2.29 | **0.02** |
| *Ramphastos culminatus* | 3 | -0.83 | 0.75 |
| *Ramphocelus carbo* | 2 | -0.30 | 0.52 |
| *Ramphastos tucanus* | 30 | -0.82 | 0.75 |
| *Selenidera reinwardtii* | 7 | -0.18 | 0.55 |
| *Tachyphonus surinamensis* | 3 | -0.14 | 0.49 |
| *Tangara callophrys* | 2 | 2.08 | **0.05** |
| *Tangara schrankii* | 6 | -0.39 | 0.64 |
| *Tangara velia* | 7 | -0.26 | 0.58 |
| *Tersina viridis* | 11 | -0.35 | 0.61 |
| *Thraupis episcopus* | 3 | 3.69 | **0.01** |
| *Trogon curucui* | 6 | -1.24 | 0.89 |
| *Trogon melanurus* | 2 | 0.46 | 0.24 |
| *Trogon rufus* | 11 | -1.24 | 0.89 |
| *Trogon viridis* | 3 | 1.19 | 0.12 |
| *Turdus albicollis* | 3 | 0.30 | 0.34 |
| *Turdus ignobilis* | 4 | 0.17 | 0.39 |
| *Tityra cayana* | 4 | -0.25 | 0.57 |

**S1 Appendix. Table D**. Patterns of phylogenetic ensemble for the frugivore species observed, according to the Nearest Taxon Index (NRI). Significantly aggregated (positive NRI values) and over-dispersed (negative) patterns are highlighted in the last column.

|  | N | NTI | P |
| --- | --- | --- | --- |
| *Lagothrix lagothricha* | 59 | 0.24 | 0.41 |
| *Ateles belzebuth* | 56 | 1.18 | 0.12 |
| *Alouatta seniculus* | 30 | 1.50 | **0.06** |
| *Sapajus apella* | 44 | -0.32 | 0.65 |
| *Saimiri cassiquiarensis* | 11 | 1.13 | 0.14 |
| *Aburria pipile* | 24 | -0.50 | 0.70 |
| *Amazona farinosa* | 9 | 0.29 | 0.39 |
| *Amazona ochrocephala* | 3 | 1.96 | **0.05** |
| *Ara macao* | 7 | 2.00 | **0.03** |
| *Ara severa* | 3 | 0.63 | 0.23 |
| *Baryphthengus ruficapillus* | 9 | -1.61 | 0.95 |
| *Brotogeris cyanoptera* | 4 | 1.38 | 0.10 |
| *Cacicus cela* | 11 | -0.46 | 0.68 |
| *Cacicus solitarius* | 3 | -0.02 | 0.40 |
| *Capito niger* | 20 | -1.16 | 0.88 |
| *Celeus elegans* | 9 | 1.12 | 0.15 |
| *Celeus flavus* | 6 | -0.79 | 0.75 |
| *Celeus grammineus* | 4 | 0.76 | 0.27 |
| *Chlorophanes spiza* | 6 | 0.06 | 0.47 |
| *Patagioena plumbea* | 7 | -1.22 | 0.90 |
| *Cyanerpes caeruleus* | 10 | -0.29 | 0.63 |
| *Cyanocorax violaceus* | 14 | -0.19 | 0.58 |
| *Dacnis cayana* | 6 | 0.42 | 0.33 |
| *Euphonia laniirostris* | 6 | 0.13 | 0.43 |
| *Euphonia minuta* | 4 | -0.32 | 0.56 |
| *Forpus conspicillatus* | 5 | 0.52 | 0.29 |
| *Gymnopithys bicolor* | 3 | -0.07 | 0.44 |
| *Gymnoderus foetidus* | 3 | -1.19 | 0.92 |
| *Gymnostinops guatimozus* | 2 | 1.98 | **0.06** |
| *Hemithraupis flavicauda* | 2 | 0.01 | 0.38 |
| *Lipaugus vociferans* | 2 | -1.07 | 0.86 |
| *Melanerpes cruentatus* | 5 | 1.55 | 0.09 |
| *Momotus momota* | 17 | 1.30 | 0.09 |
| *Penelope jaquacu* | 3 | -0.67 | 0.75 |
| *Piaya cayana* | 5 | 0.61 | 0.28 |
| *Pionites melanocephala* | 2 | 0.09 | 0.39 |
| *Pionus menstruus* | 7 | 1.69 | **0.05** |
| *Pipra coronata* | 3 | 0.12 | 0.34 |
| *Pipra erythrocephala* | 6 | 0.40 | 0.39 |
| *Pipra filicauda* | 2 | -0.33 | 0.64 |
| *Pipra sp.* | 2 | -0.33 | 0.55 |
| *Piranga olivacea* | 2 | -0.34 | 0.53 |
| *Porphyrolaema porphyrolaema* | 3 | -0.15 | 0.48 |
| *Psarocolius angustifrons* | 13 | 1.36 | 0.09 |
| *Psarocolius decumanus* | 16 | 1.33 | 0.09 |
| *Pteroglossus castanotis* | 2 | 0.08 | 0.40 |
| *Pteroglossus flavirostris* | 26 | 0.25 | 0.43 |
| *Pteroglossus pluricinctus* | 8 | 0.90 | 0.21 |
| *Pteroglossus inscriptus* | 16 | 1.27 | 0.10 |
| *Pyrrhura melanura* | 5 | 1.46 | 0.08 |
| *Querula purpurata* | 9 | 1.72 | **0.05** |
| *Ramphastos culminatus* | 3 | -0.67 | 0.74 |
| *Ramphocelus carbo* | 2 | -0.30 | 0.52 |
| *Ramphastos tucanus* | 30 | 1.23 | 0.11 |
| *Selenidera reindwardtii* | 7 | 0.32 | 0.36 |
| *Tachyphonus surinamensis* | 3 | -0.51 | 0.66 |
| *Tangara callophrys* | 2 | 1.97 | **0.06** |
| *Tangara schrankii* | 6 | -1.00 | 0.83 |
| *Tangara velia* | 7 | -0.73 | 0.74 |
| *Tersina virids* | 11 | -0.79 | 0.76 |
| *Thraupis episcopus* | 3 | 3.33 | **0.00** |
| *Trogon curucui* | 6 | -0.76 | 0.75 |
| *Trogon melanurus* | 2 | 0.38 | 0.26 |
| *Trogon rufus* | 11 | 0.75 | 0.23 |
| *Trogon viridis* | 3 | 1.04 | 0.17 |
| *Turdus albicollis* | 3 | -0.01 | 0.40 |
| *Turdus ignobilis* | 4 | -0.26 | 0.54 |
| *Tityra cayana* | 4 | -0.29 | 0.59 |

**S2 Appendix, Table S5**. Number of network indices identifying the corresponding plant species among the first 10 more important ones for the plant species studied in Tinigua National Park, Colombia.

| Species | Number of indices | Indices |
| --- | --- | --- |
| *Cecropia membranacea* | 10 | ND, WB, WC, *d', d,* NR, D, EF, SSP, G |
| *Cestrum racemosum* | 10 | ND, WB, WC, *d',* d, NR, D, EF, SSP, G |
| *Bursera inversa* | 9 | ND, WB, WC, *d',* d, NR, D, SS, G |
| *Coussapoa orthoneura* | 9 | ND, WB, WC, NR, D, SS, EP, SSP, G |
| *Laetia procera* | 9 | ND, WB, WC, NR, D, SS, EP, SSP, G |
| *Ocotea oblonga* | 9 | ND, WB, WC, NR, D, SS, EP, SSP, G |
| *Pseudolmedia hirsuta* | 9 | ND, WC, *d', d,* NR, D, EP, SSP, G |
| *Clusia palmicida* | 8 | ND, WB, NR, D, SS, EP, SSP, G |
| *Hieronyma alchorneoides* | 8 | ND, WB, NR, D, SS, EP, SSP, G |
| *Nectandra membranacea* | 8 | ND, WC, NR, D, SS, EP, SSP G |
| *Ficus davidsionii* | 7 | ND, WB, WC, NR, D, SS, G |
| *Ficus sphenophylla* | 7 | ND, WC, *d', d,* NR, D, G |
| *Henriettella fissanthera* | 7 | ND, WB, WC, NR, D, SS, G |
| *Pseudolmedia laevigata* | 7 | ND, WB, WC, NR, D, SS, G |
| *Castilla ulei* | 6 | ND, WB, WC, NR, D, G |
| *Cecropia sciadophylla* | 6 | ND, WB, WC, NR, D, G |
| *Guatteria cf. punctata* | 6 | ND, WC, NR, D, SS, G |
| *Sapium laurifolium* | 6 | ND, WC, NR, D, SS, G |
| *Trichilia tuberculata* | 6 | WB, WC, *d', d,* EP, SSP |
| *Virola calophylla* | 6 | ND, WB, SS, EP, SSP, G |
| *Maytenus cf. macrocarpa* | 5 | ND, WB, WC, NR, D |
| *Trichilia pleeana* | 4 | *d', d,* EP, SSP |
| *Virola flexuosa* | 4 | WC, NR, D, G |
| *Virola peruviana* | 4 | *d', d,* EP, SSP |
| *Alibertia cf. hadrantha* | 3 | WB, *d', d* |
| *Casearia aculeata* | 3 | SS, EP, SSP |
| *Spondias venulosa* | 3 | *d', d,* SSP |
| *Cayaponia granatensis* | 2 | *d', d* |
| *Clusia nigrolineata* | 2 | SS, EP |
| *Crepidospermum rhoifolium* | 2 | *d', d* |
| *Garcinia macrophylla* | 2 | *d', d* |
| *Helicostylis tomentosa* | 2 | *d', d* |
| *Inga acreana* | 2 | EP, SSP |
| *Inga acrocephala* | 2 | EP, SSP |
| *Inga alba* | 2 | *d', d* |
| *Leonia glycycarpa* | 2 | *d', d* |
| *Pourouma bicolor* | 2 | EP, SSP |
| *Pourouma petiolulata* | 2 | *d', d* |
| *Pouteria caimito* | 2 | EP, SSP |
| *Pouteria procera* | 2 | *d', d* |
| *Protium sagotianum* | 2 | WB, WC |
| *Pseudomalmea dielsiana* | 2 | EP, SSP |
